# Endobronchial Metastasis as the Dominant Pulmonary Manifestation of Sigmoid Colon Adenocarcinoma: A Case Report

**DOI:** 10.7759/cureus.113470

**Published:** 2026-07-27

**Authors:** Alejandra P Rivera Caro, Josean M Rosado Rivera, Rosa Roman Carlo, Milton D Carrero Quiñones, Naomi Perez

**Affiliations:** 1 Internal Medicine, Mayaguez Medical Center, Mayagüez, PRI; 2 Internal Medicine, Mayaguez Medical Center, Mayaguez, PRI; 3 Pulmonary and Critical Care, Mayagüez Medical Center, Mayagüez, PRI; 4 Internal Medicine, Mayagüez Medical Center, Mayagüez, PRI

**Keywords:** ck20, colorectal cancer, endobronchial metastasis, flexible bronchoscopy, pulmonary metastases

## Abstract

Pulmonary metastases from extrapulmonary malignancies are common; however, when a patient with a history of colorectal cancer develops new respiratory symptoms, clinicians typically look to the lung parenchyma for pulmonary metastases and rarely to the airway itself. Endobronchial metastases (EBM) occur when tumor deposits are confined to the bronchial epithelium and airway lumen rather than the surrounding lung tissue. This represents a distinct and often overlooked pattern of spread, and those originating from colorectal cancer are especially uncommon.

This report presents a case of a 60-year-old man with sigmoid colon adenocarcinoma and synchronous peritoneal metastasis (TNM stage M1c), treated with colectomy followed by adjuvant FOLFOX chemotherapy for approximately one month. Approximately two weeks after completing the first chemotherapy cycle, he developed progressive dyspnea, dry cough, dizziness, and fatigue. Chest imaging demonstrated multiple bilateral pulmonary nodules and opacities concerning for multifocal pneumonia versus lymphangitic carcinomatosis with possible endobronchial involvement, and he developed hypoxemia requiring supplemental oxygen. Despite empiric antibiotic therapy for presumed pneumonia, his symptoms persisted, prompting bronchoscopy with endobronchial and transbronchial biopsy, which revealed endobronchial lesions in the lingula and right middle lobe. Histopathology and immunohistochemistry (CDX2-positive, CK7-negative, and TTF-1-negative) were consistent with metastatic colorectal adenocarcinoma. Despite multidisciplinary management, his respiratory status progressively deteriorated, and he died of refractory respiratory failure. This case underscores that EBM should be considered in any oncology patient with persistent respiratory symptoms and pulmonary lesions, even without a parenchymal mass, and that bronchoscopy with biopsy remains the diagnostic gold standard for distinguishing EBM from primary lung cancer, sarcoidosis, and infection, and for guiding prognosis-directed and palliative management.

## Introduction

The lung is one of the most common sites for metastasis from extrapulmonary malignancies, with metastatic deposits typically located within the pulmonary parenchyma. Endobronchial metastasis (EBM) refers to tumor deposits confined to the bronchial epithelium and airway lumen rather than the surrounding lung tissue. This represents a distinct and often overlooked pattern of spread, and those originating from colorectal cancer are especially uncommon. The reported frequency of EBM varies widely across the literature, from approximately 2-36%; this range largely reflects differences in the populations studied and the diagnostic method used, since postmortem series that systematically dissect the airways of patients already known to have pulmonary metastases identify EBM far more often than autopsy or clinical series drawn from unselected patients with any solid tumor. In postmortem examinations of patients with known pulmonary metastatic disease, the incidence of EBM has been reported as high as 33%, whereas autopsy series of unselected patients dying with solid tumors report an incidence closer to 2% [1,2].

The mechanisms by which extrapulmonary tumors reach the bronchial epithelium are thought to include hematogenous seeding of the peribronchial capillary bed with subsequent centripetal growth into the airway lumen, direct extension from an adjacent parenchymal or hilar metastatic deposit, and, less commonly, retrograde lymphangitic spread along the peribronchial lymphatics [3,4]. Colorectal cancer reaches the lung predominantly through hematogenous spread via the systemic venous circulation, and this same route is thought to underlie the comparatively rare instances in which tumor cells implant within the bronchial wall itself rather than the surrounding parenchyma [2,5]. 

EBM has been most frequently described in association with breast, colorectal, and renal cell carcinoma. Colorectal cancer alone is estimated to account for 11%-29% of all reported EBM cases, a proportion that is projected to increase in parallel with the rising global incidence of colorectal cancer [2]. Reported series remain small: one French cohort described seven patients with colorectal EBM followed over several years, and isolated case reports describe EBM presenting years after primary treatment, in some instances closely mimicking sarcoidosis on both imaging and bronchoscopy [3-5]. Because isolated EBM without evidence of metastatic disease elsewhere can closely resemble primary bronchogenic carcinoma or granulomatous disease such as sarcoidosis both radiographically and bronchoscopically, it is frequently misdiagnosed at initial presentation [5].

The present case adds to this limited literature by illustrating how colorectal EBM can present acutely, with rapidly progressive hypoxemic respiratory failure and radiographic features mimicking both lymphangitic carcinomatosis and infectious pneumonia, and by directly correlating computed tomography, bronchoscopic, histopathologic, and immunohistochemical findings within a single, well-documented case. The purpose of this report is to present a case of a 60-year-old male construction worker with sigmoid colon adenocarcinoma and synchronous peritoneal metastasis (TNM stage M1c), treated with sigmoid colectomy achieving clear margins followed by adjuvant FOLFOX chemotherapy for approximately one month, who approximately two weeks after completing chemotherapy developed progressive dyspnea on exertion, dry cough, dizziness, and fatigue, and to review the relevant literature on presentation, diagnosis, and management of this rare entity.

## Case presentation

A 60-year-old male construction worker presented initially with a two-month history of progressive left lower quadrant abdominal pain and constipation. Work-up led to a diagnosis of sigmoid colon adenocarcinoma with synchronous peritoneal metastasis, staged as TNM M1c. Pathology of the primary tumor demonstrated positivity for CDX2 and CK7, and was negative for TTF-1. He underwent sigmoid colectomy with pathologically clear surgical margins, followed by adjuvant chemotherapy with FOLFOX for approximately one month. The specific histologic subtype and tumor grade of the primary lesion were not available in the records accessible to the authors.

**Figure 1:**
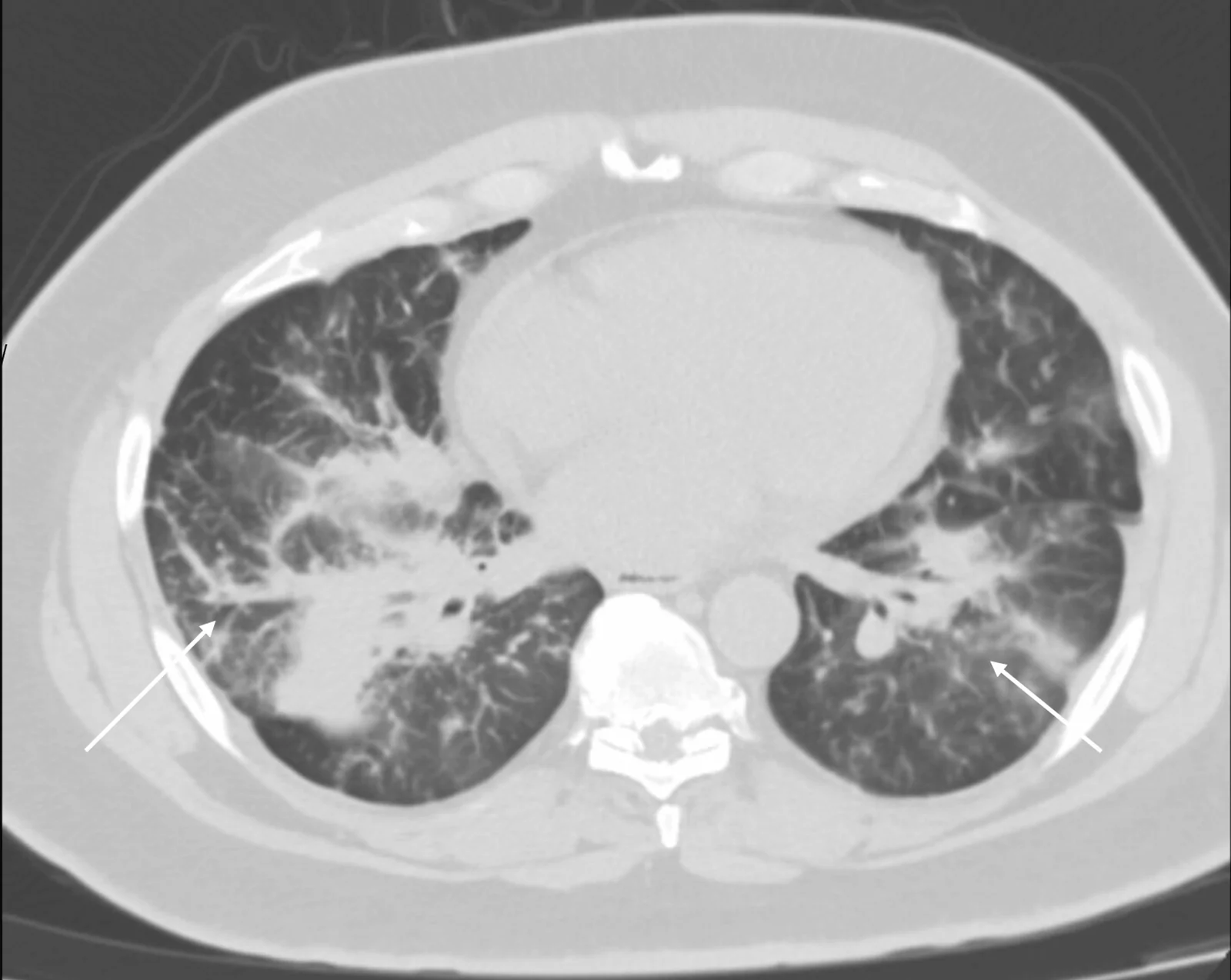
Chest computed tomography: Patchy and confluent pulmonary opacities in the lower lobes bilaterally (white arrows) with nodular interlobular septal thickening, extensive reticulation and groundglass opacities throughout both lungs.

Approximately two weeks after completing the first cycle of chemotherapy, the patient developed progressive dyspnea on exertion, a persistent dry cough, dizziness, and marked fatigue. Chest computed tomography revealed multiple bilateral pulmonary nodules with associated opacities (Figure 1), initially suggestive of multifocal pneumonia, but possible endobronchial metastatic involvement was also considered; this radiographic impression was based on the pattern of nodular and reticular opacities on CT in the context of known malignancy. His clinical course was complicated by worsening hypoxemia requiring supplemental oxygen, and he was initially treated empirically with intravenous antibiotics for presumed healthcare-associated pneumonia without significant improvement.

Baseline laboratory evaluation showed a white blood cell count of 6.83 ×10³/µL, hemoglobin 13.3 g/dL, hematocrit 39.3%, and platelet count 185 ×10³/µL, with a procalcitonin of 0.142 ng/mL. Liver function testing showed AST 135 U/L and ALT 341 U/L, with alkaline phosphatase 105 U/L. Carcinoembryonic antigen (CEA) was not obtained. Further blood cultures were negative.

Given his persistent respiratory symptoms, laboratory values (Table 1), radiographic findings, and known malignancy history, he was transferred for pulmonology evaluation and underwent diagnostic flexible bronchoscopy with endobronchial and transbronchial biopsy. The precise interval between symptom onset and bronchoscopic diagnosis could not be determined from the records available to the authors.

**Table 1:** Baseline laboratory values on presentation, with institutional reference ranges.

Laboratory Test	Patient Value	Reference Range
White blood cell count	6.83 ×10³/µL	4.5–11.0 ×10³/µL
Hemoglobin	13.3 g/dL	13.5–17.5 g/dL (male)
Hematocrit	39.3%	38.8–50.0% (male)
Platelet count	185 ×10³/µL	150–450 ×10³/µL
Procalcitonin	0.142 ng/mL	<0.05 ng/mL
AST	135 U/L	8–48 U/L
ALT	341 U/L	7–55 U/L
Alkaline phosphatase (ALP)	105 U/L	40–129 U/L
Carcinoembryonic antigen (CEA)	Not obtained	≤3.0 ng/mL (nonsmoker)

Flexible bronchoscopy was performed using an Olympus flexible video bronchoscope. The following procedures were performed with fluoroscopic guidance: bronchial and endobronchial biopsy of the lingula; transbronchial lung biopsy of a single lobe (right middle lobe); bronchoalveolar lavage; diagnostic cell washing; and brushing/protected brushings.

On systematic airway examination, the vocal cords were of normal appearance with mild bowing of the mid-cord region, and the false cords were noted to be redundant. Diffuse tracheobronchomalacia was present, with a fixed carina. The left bronchial tree demonstrated mucosal edema and a color change at the lingular segment of the left upper lobe, with concentric narrowing of the luminal diameter. The right bronchial tree showed edematous mucosa with a decreased luminal diameter of the right middle lobe bronchus due to extrinsic compression, which collapsed easily and could not be fully opened; no definitive endobronchial lesion was visualized on the right. Biopsies were obtained from the endobronchial lesion at the lingular segment and from the right middle lobe via transbronchial biopsy.

**Figure 2:**
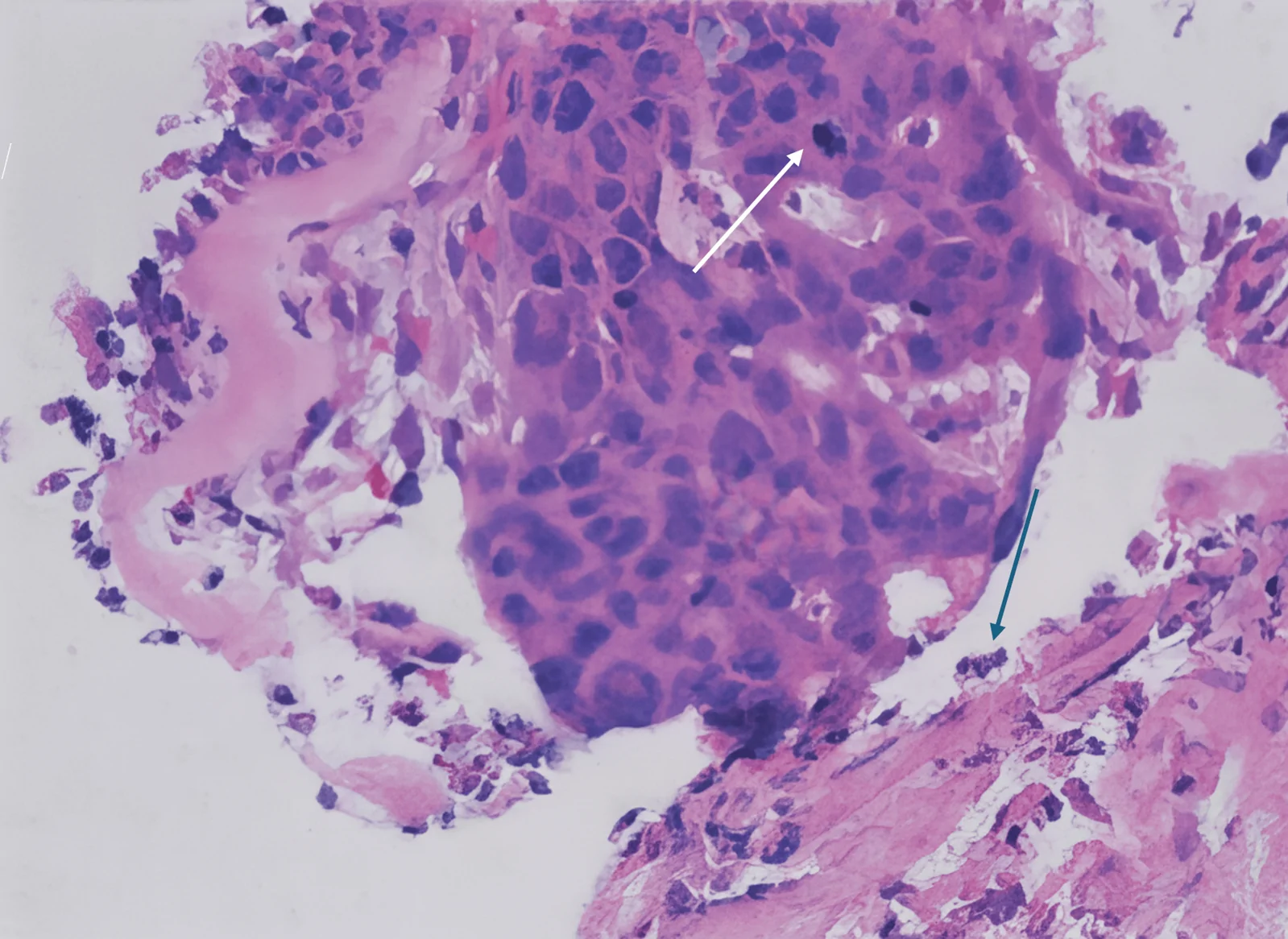
Immunohistochemical staining of the lingular endobronchial biopsy, original magnification 200x. (A) CDX2 demonstrates strong nuclear positivity in tumor cells (arrow-white). (B) CK7 is negative in tumor cells (arrow-blue), with an internal positive control seen in adjacent normal bronchial epithelium. Together, this CDX2-positive/CK7-negative immunophenotype supports a colorectal, rather than primary pulmonary, origin of the tumor. TTF-1 immunohistochemistry was also performed and was negative in tumor cells, arguing against a primary pulmonary origin; a corresponding photomicrograph was not available for inclusion. Together, this CDX2-positive/CK7-negative/TTF-1-negative immunophenotype is supportive of a colorectal, rather than primary pulmonary, origin of the tumor.

Histopathologic examination of both specimens demonstrated infiltrating adenocarcinoma (Figure 2). Immunohistochemical staining showed a CDX2-positive, CK7-negative, TTF-1-negative profile (Figure 2); SATB2 was not performed. This immunophenotype is strongly supportive of, though not independently diagnostic of, a colorectal rather than primary pulmonary origin: the CDX2-positive pattern is characteristic of gastrointestinal differentiation, while the negative TTF-1 argues against a primary lung adenocarcinoma, which is TTF-1-positive in most cases. This immune profile was interpreted in conjunction with the patient's known history of colorectal adenocarcinoma rather than in isolation. The endobronchial lingular biopsy and the right middle lobe transbronchial biopsy were both reported as positive for metastatic adenocarcinoma consistent with a colonic primary. Archived tissue from the primary sigmoid resection was not available for direct side-by-side histologic comparison; however, the original pathology report for the primary tumor documented positivity for CDX2 and CK7. The pulmonary biopsies obtained during this admission shared CDX2 positivity with the primary tumor report but differed in CK7 status (negative in the pulmonary biopsies versus positive reported for the primary tumor); this discordance is discussed further below.

The patient remained hospitalized on supplemental oxygen and was managed by a multidisciplinary team including Pulmonology, Infectious Disease, Surgery, and Hematology/Oncology. Despite these efforts, his respiratory status progressively declined, and he died of unresolved respiratory failure while on high-flow nasal cannula support several weeks after the diagnosis of EBM was established.

## Discussion

Pulmonary metastases from colorectal cancer most commonly present as multiple, well-circumscribed parenchymal nodules identified on surveillance or symptom-driven imaging. Endobronchial involvement, by contrast, is a comparatively rare pattern of spread that has been described with several primary malignancies, including breast, renal cell, and colorectal carcinoma [1,2]. Reported incidence figures for EBM vary considerably depending on study design, as discussed above: postmortem series restricted to patients with known pulmonary metastatic disease have reported EBM rates as high as 33%, while broader autopsy series of patients dying with solid tumors of any type report rates closer to 2% [2-4].

Regardless of the underlying mechanism of spread, the clinical consequence of EBM is a lesion that behaves, both radiographically and bronchoscopically, much like a primary endobronchial tumor. Patients with EBM most commonly present with cough, dyspnea, hemoptysis, recurrent or non-resolving pneumonia due to post-obstructive changes, or, as in this case, progressive hypoxemia [2,3]. Because these findings overlap substantially with primary bronchogenic carcinoma, granulomatous disease such as sarcoidosis, and infectious processes, EBM is frequently misdiagnosed, or diagnosis is delayed until bronchoscopic tissue sampling is performed [5].

Bronchoscopy with biopsy remains the diagnostic procedure of choice, allowing direct visualization of the endobronchial lesion and tissue acquisition for histopathologic and immunohistochemical confirmation. In the present case, an immunophenotype of CDX2 positivity and CK7 negativity was strongly supportive of a colorectal primary, distinguishing this lesion from a primary lung adenocarcinoma, which is typically CK7-positive and CK20-negative. This immunophenotype alone is not independently diagnostic; however, it was interpreted alongside the patient's known history of colorectal adenocarcinoma rather than in isolation. Notably, the primary tumor's pathology report described CK7 positivity, in contrast to the CK7-negative pulmonary biopsies. Discordant CK7 expression between primary and metastatic colorectal lesions has been described in the literature and likely reflects intratumoral phenotypic heterogeneity rather than a distinct clonal origin; the concordant CDX2, together with the patient's clinical history and the morphologic appearance of the lesion, remained most consistent with metastatic disease from the known colorectal primary. This distinction is clinically important, since the differential diagnosis at bronchoscopy includes primary lung cancer, sarcoidosis, and metastatic disease and carries markedly different prognostic and therapeutic implications [3,5]. Compared with previously reported colorectal EBM cases, which most often describe lesions in the lower lobe or mainstem bronchi, involvement of the lingular segment in the present case is a somewhat atypical location, though the small size of published series limits any firm conclusions about anatomic predilection [2,6].

Management of EBM is generally guided by the extent of disease and the patient's overall clinical status. Systemic chemotherapy directed at the underlying primary tumor remains the mainstay of treatment for patients with disseminated disease; in the present case, the patient already carried a diagnosis of metastatic (TNM M1c) colorectal adenocarcinoma with peritoneal involvement at the time of initial diagnosis, and pulmonary and endobronchial involvement emerged shortly after completing a first course of FOLFOX chemotherapy, reflecting disease progression in an already advanced-stage malignancy rather than a late recurrence after apparently curative treatment. For patients with localized, symptomatic airway obstruction, interventional bronchoscopic techniques including mechanical debulking, laser ablation, cryotherapy, endobronchial stenting, or external-beam radiotherapy can provide meaningful palliation of dyspnea, cough, and post-obstructive infection, and have been shown to improve quality of life even when disease-modifying therapy is limited [2]. Unfortunately, in patients who present with diffuse bilateral parenchymal and endobronchial involvement, as illustrated here, the prognosis remains poor, and respiratory failure may progress despite multidisciplinary supportive care.

This case reinforces several points emphasized in the existing literature. First, EBM should remain on the differential diagnosis for any patient with a history of colorectal cancer who develops new or progressive respiratory symptoms, even when initial imaging is more suggestive of lymphangitic carcinomatosis or infectious pneumonia than of a discrete endobronchial mass [1,4]. Second, empiric treatment for infection should not delay bronchoscopic evaluation when symptoms persist despite antimicrobial therapy, since earlier tissue diagnosis may allow for earlier initiation of systemic or palliative airway-directed therapy. Third, as illustrated in prior reports of EBM masquerading as sarcoidosis, reliance on radiographic pattern alone is insufficient, and histopathologic and immunohistochemical confirmation is required in every case [5].

In this case, bronchoscopy was pursued only after an empiric course of antibiotics for presumed pneumonia failed to produce clinical improvement. Whether earlier bronchoscopic evaluation, performed at initial presentation with pulmonary nodules and opacities rather than after a trial of empiric antibiotics, would have altered the patient's ultimate prognosis is uncertain, given the bilateral, multifocal nature of the parenchymal disease already present on initial imaging and the patient's already-established metastatic (M1c) disease burden. However, earlier diagnosis may have allowed for earlier initiation of next-line systemic therapy directed at the progressing metastatic colorectal cancer, and potentially earlier consideration of local airway-directed palliation of the lingular obstruction, before the patient's respiratory status had deteriorated to the point of requiring high-flow oxygen support. This underscores a broader principle: in oncology patients with persistent respiratory symptoms that do not respond to empiric antimicrobial therapy, bronchoscopic evaluation should not be delayed, since earlier tissue diagnosis is the modifiable step most likely to influence subsequent management [2,3].

Published outcomes for EBM specifically arising from colorectal cancer are limited to small case series, but they consistently describe a poor overall prognosis once endobronchial involvement is identified. In one series of seven patients with colorectal EBM, median survival after the diagnosis of EBM was 18.9 months; notably, in that same cohort, patients diagnosed with EBM had a longer median survival from the time of pulmonary metastasis diagnosis than a comparison group with parenchymal-only pulmonary metastases (55.7 versus 12.7 months), a finding the authors attributed to earlier detection prompted by symptomatic airway involvement [6]. The present case illustrates a more aggressive trajectory since the patient already had established metastatic (M1c) disease at the time of his original colorectal cancer diagnosis, and pulmonary and endobronchial progression occurred within weeks of completing initial chemotherapy, with death from respiratory failure only several weeks after the diagnosis of EBM. This underscores that outcomes in colorectal EBM remain highly variable and are likely driven as much by the extent of associated parenchymal and systemic disease as by the endobronchial lesion itself.

## Conclusions

EBM is an uncommon but clinically significant manifestation of colorectal adenocarcinoma that can mimic primary lung malignancy, sarcoidosis, or infectious pneumonia. This case underscores the importance of maintaining a high index of suspicion for EBM in patients with a history of colorectal cancer who present with persistent or progressive respiratory symptoms, particularly when imaging demonstrates pulmonary nodules, opacities, or findings suggestive of airway involvement. Bronchoscopic tissue biopsy with immunohistochemical characterization remains essential for accurate diagnosis and for distinguishing EBM from primary pulmonary disease; however, because the present single-patient case report cannot establish that earlier bronchoscopic evaluation changes prognosis, this should not be inferred from the present report. Rather, what this case illustrates is a modifiable point in management, namely that bronchoscopic evaluation should not be delayed pending resolution of empiric antibiotic therapy in an oncology patient with persistent respiratory symptoms, since tissue diagnosis is the step most likely to influence subsequent treatment decisions.
